# EpiHacks, a Process for Technologists and Health Experts to Cocreate Optimal Solutions for Disease Prevention and Control: User-Centered Design Approach

**DOI:** 10.2196/34286

**Published:** 2021-12-15

**Authors:** Nomita Divi, Mark Smolinski

**Affiliations:** 1 Ending Pandemics San Francisco, CA United States

**Keywords:** epidemiology, public health, diagnostic, tool, disease surveillance, technology solution, innovative approaches to disease surveillance, One Health, surveillance, hack, innovation, expert, solution, prevention, control

## Abstract

**Background:**

Technology-based innovations that are created collaboratively by local technology specialists and health experts can optimize the addressing of priority needs for disease prevention and control. An EpiHack is a distinct, collaborative approach to developing solutions that combines the science of epidemiology with the format of a hackathon. Since 2013, a total of 12 EpiHacks have collectively brought together over 500 technology and health professionals from 29 countries.

**Objective:**

We aimed to define the EpiHack process and summarize the impacts of the technology-based innovations that have been created through this approach.

**Methods:**

The key components and timeline of an EpiHack were described in detail. The focus areas, outputs, and impacts of the twelve EpiHacks that were conducted between 2013 and 2021 were summarized.

**Results:**

EpiHack solutions have served to improve surveillance for influenza, dengue, and mass gatherings, as well as laboratory sample tracking and One Health surveillance, in rural and urban communities. Several EpiHack tools were scaled during the COVID-19 pandemic to support local governments in conducting active surveillance. All tools were designed to be open source to allow for easy replication and adaptation by other governments or parties.

**Conclusions:**

EpiHacks provide an efficient, flexible, and replicable new approach to generating relevant and timely innovations that are locally developed and owned, are scalable, and are sustainable.

## Introduction

Technology can play an important role in the prevention and control of infectious disease. Historically, developing technical solutions has required substantial financial resources, human capacity, and time. All too often, innovative technologies that are introduced as pilot projects or research endeavors are terminated prematurely due to a lack of sustained funding or a lack of a postresearch plan. Third-party technologies that are created for governments are often discarded when vendors change or fail to create long-term plans for sustainability and scaling. In other cases, misalignment with current contexts and capabilities has often resulted in technologies being unsuccessful despite the best intentions. In short, technology solutions that are created *for* low- and middle-income countries to address local challenges, rather than those created *by* such countries, are quickly becoming a thing of the past.

Creating scalable and sustainable innovations for disease prevention and control requires local technologists to work together with health experts to optimize the desired results when creating technology-based innovations. Conducting an EpiHack is one way to optimize such results. EpiHacks provide a unique process for combining the science of epidemiology with elements of a hackathon to cocreate solutions that address existing and future needs through a highly collaborative and intensive event.

Engaging local technologists and health experts from idea inception to prototype development can ensure that the appropriate contexts, such as language, culturally relevant imagery, and social norms, are duly considered when creating any solution to identified challenges. Additionally, innovative solutions that are generated through the use of existing infrastructures and widely available technologies are more likely to be widely adopted and affordable.

In an EpiHack, design thinking is the principal approach that is used to identify needs and guide the development of tools and systems to meet those needs. Design thinking is a process for solving problems by prioritizing end users’ needs through observing how people interact with their environments and by using an iterative, hands-on approach to creating innovative solutions [1]. The focus of an EpiHack can be specifically on disease, situations, or improved data analysis and visualization. EEpiHack solutions have served to improve surveillance for influenza, dengue, and mass gatherings, as well as laboratory sample tracking and One Health surveillance, in rural and urban communities. As of January 2021, a total of 12 EpiHacks have been executed across 5 continents, resulting in open-source technologies positively impacting disease prevention and control. This paper defines the EpiHack process and summarizes the impacts of the technology-based innovations that have been created through this approach.

## Methods

EpiHack elements and the timeline of an EpiHack were described. The twelve EpiHacks conducted between 2013 and 2021 were reviewed to document the focus areas, outputs, and impacts.

### EpiHack Components and Timeline

**Figure 1:**
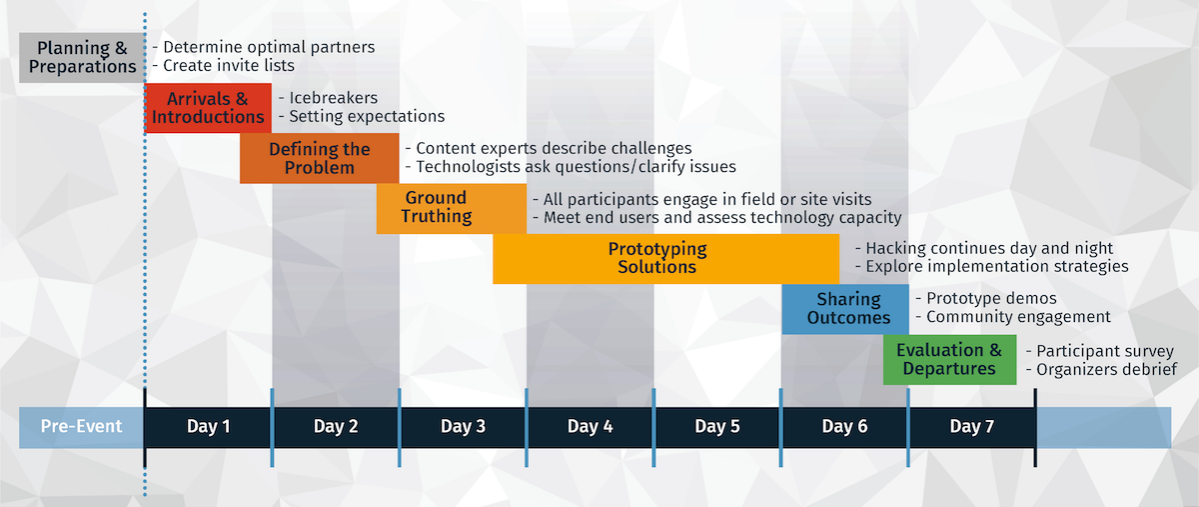
EpiHack components and timeline. EpiHacks are ideally conducted over a 1-week period to provide optimum time for defining the problems, exploring solutions, and arriving at prototypes by the conclusion of the event.

Each EpiHack prioritized the needs of the host country or organization to solve real-world problems efficiently and effectively through a highly interactive and collaborative process. EpiHacks are immersive, residential programs in which developers and health experts work side by side on-site until the desired outcomes are achieved. EpiHacks generally occur over the period of 1 week but can be adapted according to the complexity of the problem(s) to be solved (Figure 1). In theory, it may be possible to host a web-based EpiHack, although this approach has not yet been tested. Participants do not compete for prizes or awards; rather, they collaborate to achieve common goals and maximize the desired impact.

### Planning and Preparation

An EpiHack requires rigorous advanced planning, including securing a location for hosting an immersive, potentially weeklong event with extended hours each day. A good deal of time and effort goes into identifying participants, which include key stakeholders from the government, the private sector, and the community along with technologists and facilitators, who are critical to ensuring the successful outcomes of the event. It is imperative that various levels of government be brought into the planning process at the earliest stage, given their critical roles and responsibilities in national and local disease prevention and control.

The host organization is typically an in-country partner who is eager to improve local disease prevention and control capacities and fill existing gaps. Host organizations have included government agencies, universities, and not-for-profit organizations. The host must oversee all planning and the execution of the event and ensure that the right mix of health and technology participants will be in attendance. An appropriate balance of subject matter expertise and technology development skills is crucial to inspiring unbridled creativity. A developer to health expert ratio of 3:1 is recommended.

Given that 3 in 4 emerging infectious diseases have their origins in animals, it is imperative that technology-enabled solutions for disease prevention and control engage both the human and animal sectors [2]. EpiHacks therefore require the active engagement of subject matter experts across the human and animal health sectors, including epidemiologists, veterinarians, and health care specialists. They also engage diverse end users, such as farmers, market workers, community health volunteers, district health officers, and lab technicians. EpiHacks also require the participation of a diverse set of local technology professionals who are familiar with front-end development (user interfaces and web design), back-end development (servers and databases), and data science elements (data management and analytics). Local technology expertise promotes mindfulness toward how proposed innovations can manifest into readily usable technologies.

Ensuring that strategic governmental partners, potential funders, and key community organizations are engaged in the planning of an EpiHack also improves the likelihood of successful outcomes. This also allows for the longer-term engagement of stakeholders from key implementing organizations, who are necessary for fully deploying the tools and systems that are created during an EpiHack.

In some cases, hosts may work with stakeholders in advance to frame the challenges that will be addressed during an EpiHack before the start of the event. In most instances however, the true nuances of these challenges come to light only after an EpiHack begins. Many aspects of an EpiHack are intentionally made to be unconfined to provide participants with the freedom to arrive at the most creative and appropriate solutions. Part of the initial planning includes arrangements for “field trips” or site visits to explore technical capabilities and to meet the potential end users of the innovations to be created during an EpiHack.

Most EpiHack attendees—both technology and health expert participants—reside in the host country. This ensures that participants are aware of the local contexts and challenges related to developing compelling prototypes that resonate with the targeted end users. Attracting and identifying the right local technology partners often require posting about the upcoming EpiHack on various media outlets and recruiting them from both the private and public sectors. Health expert attendees are generally invited based on their area of expertise or role within the government with regard to disease prevention and control.

### Arrivals and Introductions

Given that many EpiHack participants do not know each other or have not attended a prior EpiHack, it is important to provide adequate time for getting acquainted through icebreakers, providing an introduction to the process of an EpiHack, and setting expectations for the days of the EpiHack. Often, the human and animal health experts, even if they are from the same country, do not know each other well and appreciate the opportunity to socialize before the intensive EpiHack process ensues. Building mutual trust and respect between the health experts and the technologists is essential, given the rapid pace of the event.

### Defining the Problem

In most EpiHacks, participants begin their substantive work by defining the problem or clarifying the challenges to be addressed over the course of the week. This focus at the start provides the opportunity for content experts to explain existing challenges and allows for technology participants to ask questions to the health experts and vice versa. In cases where the problem to be addressed is determined through pre-event work, the EpiHack starts off with the host organization updating the participants on the desired goals.

### Ground Truthing

Once the challenges have been defined, the participants travel to the “field” to visit relevant sites, so that they can better understand the needs of end users. During these visits, participants interact directly with multiple end users of the proposed technology solution(s) and explore their operating environments. This may include visits to government facilities, community health clinics, hospitals, or live animal markets to examine the existing workflows and current infrastructures with which the proposed tools will operate. These visits might entail engaging with farmers in rural villages or observing community health workers in their catchment areas to understand firsthand the challenges to be resolved. EpiHack solutions must be capable of integration and interoperation with existing systems and use the pervasive technologies in a given country or region. These solutions must also account for issues, such as those related to internet connectivity and ease of use.

### Prototyping Solutions

An important part of an EpiHack is hacking, and this takes place over the several days of the so-called *hacking stage*. Participants work collaboratively to iterate on ideas, test concepts by developing mock-ups, and begin to create functional prototypes. During this time, technology experts work closely with their health counterparts to resolve issues related to design, testing, and strategies for implementation. Although hacking is the primary function of the technologists, the health experts use this time to further develop plans for pilot tests, end-user engagement, and the scaling of the solutions. Often, the participants divide themselves into smaller teams to concentrate on building a specific tool or to allow for intense focus on a particular issue.

All tools, prototypes, and systems that are developed in EpiHacks are open source, meaning that the software for original source codes is made freely available and may be modified. This allows others to replicate or adapt the tools for their own purposes. Participants are asked to sign an open-source agreement to this effect, waiving any intellectual property rights for the ideas or tools that are developed during EpiHack events. The designs, prototypes, and tools that are created during an EpiHack are typically further developed into fully deployed systems that serve the host country or organization following the event. In many cases, the collaborations among the technologists and health experts that occur during an EpiHack continue well after the week’s end.

### Sharing Outcomes

The penultimate event of the week is the showcasing of the solutions and prototypes developed during the EpiHack among the participants and key stakeholders. Community members are also invited to this event to broaden engagement and further facilitate the uptake of the solutions. Private sector participation can often help with devising strategies and creating necessary partnerships for financing and scaling tools. Such partnerships are especially useful when large-scale health emergencies require the rapid adoption and widespread use of solutions.

### Evaluation and Departures

The work of an EpiHack does not end after participants share the outcomes. Often before leaving, participants make commitments to certain outcomes and align their roles with those of other stakeholders to ensure the completion and sustainability of the proposed solutions. Many times, participants create a mailing list or a WhatsApp group to update each other on the progress of tool development post-EpiHack and to share opportunities for further engagement. At the conclusion of each EpiHack event, participants complete a survey, and organizers hold an after-action debrief to evaluate the impact of the EpiHack and identify areas for improvement.

### Research Ethics Statement

This study did not receive or require ethics approval, as it did not involve human or animal subjects.

## Results

Since 2013, a total of 12 EpiHacks have been conducted, and each event resulted in 1 or more prototypes of tools or systems for enhanced disease prevention and control (Table 1).

EpiHack solutions have included national hotlines, One Health participatory surveillance systems, health monitoring tools for mass gatherings, and systems for mosquito vector control. EpiHack Phnom Penh, for example, laid the groundwork for the 115 Hotline—an interactive voice response system that allows Cambodians nationwide to report on suspected health threats in their community as well as access a menu of health education messages [3,4]. During the COVID-19 response, this free national hotline became the primary means for reporting COVID-19 suspect cases, initiating contact tracing efforts, and sharing COVID-19–related health information to the community. The hotline received an average of 18,000 calls per day at the peak of the COVID-19 pandemic and an average of 600 calls per day before the pandemic [5].

EpiHack Chiang Mai resulted in the Participatory One Health Disease Detection (PODD) system in Thailand, which uses smartphone apps and web applications to empower trained village volunteers to report unusual disease events in livestock, wild animals, and humans. By using this system, reports are triaged by PODD analysts and result in a response from local public health and livestock offices when warranted [6,7]. To date, over 19,000 volunteers have been trained to use the PODD system, and the project has expanded to approximately 400 subdistricts across 27 provinces in Thailand. In addition, community members used the PODD system to track the health of nonresidents who entered their village during the government-instituted COVID-19 lockdowns. Among 340 applications from 61 countries, the PODD system was the grand prize winner of the Trinity Challenge—an initiative that was created by a coalition of companies, foundations, and universities to recognize innovations in data-driven research and analytics for global health emergencies [8].

EpiHack Arusha participants created digital prototypes that resulted in the AfyaData mobile app for One Health surveillance in Tanzania. The system is used by trained volunteers in the Morogoro and Ngorongoro districts in Tanzania and allows for the real-time collection of data on human and animal health. AfyaData collects information at both the community and health facility levels and returns information on potential health intervention strategies to the reporters [9]. During the COVID-19 pandemic, AfyaData was shared with Mozambique for the cross-border tracking of COVID-19.

Due to the successful use of participatory surveillance for the first time at a mass gathering during the 2014 World Cup in Brazil, EpiHack Rio de Janeiro hosted a diverse set of international participants in 2015 to replicate this surveillance approach and enable the early identification of disease clusters during the Rio Olympic and Paralympic Games [10,11]. Members from India attended this EpiHack and incorporated their learnings from mass gathering disease surveillance during religious events in India [12]. The participatory surveillance tool in Brazil has since been adapted to monitor symptom reports from the public in Brazil and to capture data on COVID-19 symptom reporting in Brazil, Chile, and Peru.

**Table 1:** Summary of the EpiHacks conducted between 2013 and 2018.

Date	Location	Participants, n	Hosts	Focus area	Output and impact
August 2013	Phnom Penh, Cambodia	55	Royal University of Phnom Penh and Cambodia Ministry of Health	Participatory surveillance	The 115 national reporting hotline was created and deployed nationwide for disease reporting by the community and relaying health alerts. Averaging 600 calls per day, the hotline scaled during the COVID-19 pandemic to 18,000 calls per day and captured over 90% of the initial COVID-19 suspect cases [5].
March 2014	Chiang Mai, Thailand	49	Chiang Mai University College of Veterinary Medicine and Chiang Mai Provincial Public Health & Livestock Offices	One Health participatory surveillance	PODD^a^ mobile apps and web-based applications were created. The PODD tool expanded from a single province and was adopted by >400 local governments across 27 provinces in Thailand with >19,000 trained volunteers [5]. During the COVID-19 pandemic, the PODD tool was adapted for tracking suspected COVID-19 cases.
June 2014	Lao People’s Democratic Republic	62	Mekong Basin Disease Surveillance Network and Lao People’s Democratic Republic Ministry of Health	Dengue surveillance	A dengue larvae survey tool was developed to allow for active community engagement in reducing the number of larvae breeding sites to improve dengue prevention and control.
December 2014	Arusha, Tanzania	74	Southern Africa Center for Infectious Disease Surveillance, Ministry of Health, and Ministry of Livestock and Fisheries	One Health participatory surveillance	The AfyaData One Health surveillance tool was developed, and it collected over 12,000 reports of abnormal health events in humans and animals over first 5 years of operation. The timeliness of local reporting to authorities improved from an average of 10 days to an average of 3 days [9]. During the COVID-19 pandemic, AfyaData was shared with Mozambique for cross-border reporting.
July 2015	Rio de Janeiro, Brazil	49	Brazil Ministry of Health	Mass gathering surveillance	The Guardians of Health surveillance tool was created, and it demonstrated the utility of participatory surveillance for monitoring health events during mass gatherings [10,11]. The tool has been used for COVID-19 surveillance among subpopulations in Brazil, Chile, and Peru.
September 2015	Minneapolis, United States of America	30	University of Minnesota Food Protection and Defense Institute and National Association of City and County Health Officials	Influenza surveillance	A Flu Near You (influenza-like illness symptom reporting system) data dashboard was created to improve data visualization, and it enhanced data analytics for use by local health departments.
April 2016	Saranda, Albania	41	Southeast European Center for Surveillance and Control of Infectious Diseases, Ministry of Health, Institute of Public Health, and Ministry of Animal Health	One Health surveillance systems	A digital data dashboard for One Health surveillance was built based on the learnings from the Thailand and Tanzania EpiHacks.
April 2016	Yangon, Myanmar	44	Myanmar Ministry of Health	Participatory surveillance	A hotline for citizen volunteers to report on health issues was developed for direct community reporting on health issues.
September 2016	Denver, United States of America	25	Council of State and Territorial Epidemiologists	Influenza surveillance	A Flu Near You data validation toolkit and data dashboard enhancements were developed for local health departments to better interpret Flu Near You data [13].
October 2017	Hanoi, Vietnam	31	Vietnam Ministry of Health General Department of Preventive Medicine	Participatory surveillance	A community outbreak reporting hotline was created by replicating the 115 Hotline model from Cambodia for use in Vietnam [14].
November 2017	Colombo, Sri Lanka	90	Colombo Municipal Council and Nanyang Technology University	Vector-borne disease surveillance	The Mo-Buzz dengue surveillance app was enhanced to improve the user interface, digitize paper forms, and improve data analytics and the visualization of breeding sites that were indicated as high-risk areas [15].
April 2018	Kampala, Uganda	44	Uganda Ministry of Health	Emergency Operations Center	A contact tracing prototype and laboratory sample tracking system were created.

^a^PODD: Participatory One Health Disease Detection.

EpiHacks have also contributed to building a community of practice around innovations in surveillance. In several instances, members who have attended an EpiHack apply their learnings to subsequent EpiHacks and new tool development. For example, health experts and developers from Tanzania attended EpiHack Chiang Mai (conducted in March 2014) to learn about the EpiHack process. Subsequently, they applied their learnings to the EpiHack in Tanzania (conducted in December 2014). Conversely, participants from EpiHack Chiang Mai attended the EpiHack in Tanzania to help guide the event. Furthermore, learnings from the Tanzania EpiHack informed the Albania EpiHack, which resulted in the development of the Albania One Health surveillance system.

EpiHack participants’ evaluations uncovered key thematic areas, including the value of dedicating time to networking, the utility of the “field trips,” and the desire for ongoing engagement with coparticipants. As noted in the evaluations, the provision of adequate time during each EpiHack for networking is prioritized. Additionally, up to 1 whole day is dedicated to the “field trip,” so that all participants can experience the realities that are relevant to their solutions. Finally, given the interest of participants in continuing their engagement after EpiHack events, email listservs and WhatsApp groups are created by the organizers to facilitate ongoing communication among participants.

## Discussion

EpiHacks provide an efficient and effective approach for generating relevant and timely innovations in disease prevention and control that are locally developed and owned. EpiHacks are well suited for tackling challenges that require creativity, consensus among stakeholders, and cross-sectoral collaboration. Political support and buy-ins from local leadership are foundational for a successful EpiHack. Although not all EpiHack hosts are government agencies, their participation is important for fostering the necessary buy-ins for the development of prototypes and sustainability.

EpiHacks offer opportunities for new collaborations and networking among participants at the national level and on a global scale. Most EpiHack participants are chosen by organizers from the host nation in keeping with a focus on local and sustainable solutions. Engaging EpiHack alumni, who understand the process due to their previous experiences, is highly beneficial. Those who are interested in hosting a future EpiHack might attend an event to experience this approach prior to hosting their own convening.

EpiHacks have been used primarily in the context of innovations for the prevention and control of infectious disease. This approach however could serve as a model for the advancement of solutions to other global health challenges. Web-based training on the EpiHack approach is free, and the health community is encouraged to consider this efficient method for advancing technology solutions to challenges in a variety of contexts [16]. The lessons gained from EpiHacks to date are that no community is too hard to reach, no country is too resource poor to innovate, and curiosity outshines fear around the globe when people are given the opportunity to create local solutions to pressing problems.

## Abbreviations

InSTEDD: Innovative Support to Emergencies Diseases and Disasters
PODD: Participatory One Health Disease Detection
